# Microstructure-Forming Mechanism of Optical Sheet Based on Polymer State Transition in Injection-Rolling Process

**DOI:** 10.3390/polym13020181

**Published:** 2021-01-06

**Authors:** Yanfeng Feng, Yan Lou, Jun Shen

**Affiliations:** 1 Guangdong Provincial Key Laboratory of Micro/Nano Optomechatronics Engineering, College of Mechatronics and Control Engineering, Shenzhen University, Shenzhen 518060, China; yffeng0209@szu.edu.cn (Y.F.); junshen@szu.edu.cn (J.S.); 2 Key Laboratory of Optoelectronic Devices and Systems of Ministry of Education and Guangdong Province, College of Physics and Optoelectronic Engineering, Shenzhen University, Shenzhen 518060, China

**Keywords:** injection-rolling, optical sheet, polymer state transition, microstructure forming mechanism

## Abstract

Polymeric optical sheets are significant components in large-scale display devices and are difficult to fabricate due to small size and high accuracy of large-area microstructures. As a newly developed molding technique, injection-rolling is capable of continuously and efficiently achieving large-area microstructures on the polymer surface with short time and high replication. However, the microstructure-forming mechanism during the injection-rolling process has not been fully understood. In this paper, a three-dimensional steady-state heat-flow coupling simulation model of the injection-rolling zone was established to obtain the distributions of the polymer state transition interfaces. According to the state transition interfaces, the entire microstructure-forming process was numerically simulated by dividing into filling and embossing stages to systematically analyze the effects of the polymer state transition interface on filling rate. After this, the relationship between process parameters such as injection temperature, rolling speed, and roll temperature and polymer state transition interface was investigated to develop a position prediction model of the state transition interface. In addition, the optical sheet injection-rolling experiments were also carried out to reveal that the filling rate of the microstructures on the optical sheet can be affected by varying the positions of the state transition interfaces. Therefore, the microstructure-forming mechanism could be revealed as theoretical guidance for the subsequent injection-rolling production with high quality and high efficiency.

## 1. Introduction

Recently, optical sheets have been intensively utilized in the large-scale liquid crystal display devices due to their excellent functions, such as guiding the light propagation path and turning a line or point light source into a uniform surface light source [1,2]. Molding technology of optical polymer microstructures has become a hot issue in this research field to rapidly and environmentally fabricate optical sheets with high quality and large volume [3,4]. The profile and distribution accuracy of the microstructures on the large-area optical sheet directly affect its optical performance and need to be strictly guaranteed. However, the precision fabrication of optical sheet with high quality, high efficiency and mass production is still a challenge, basically due to the limited efficiency and machining precision of the existing molding technologies. Therefore, scholars have conducted a lot of research on molding methods for optical sheet fabrication, such as imprinting, injection molding and micro hot embossing method, etc. [5,6,7].

So far, a common approach for large-volume optical sheet fabrication is to machine the micro/nanostructures on the template surface, and then replicate the structures onto the polymer surface, named as micro/nano-imprinting [8]. The imprinting of polymer microstructures mainly includes flat-plate hot imprinting and hot rolling [9]. Scholars such as Kolli [10] and Wu [11] have studied the plate-to-plate (P2P) isothermal imprinting method, which has the advantages of good replication, low production cost and high molding efficiency, yet has difficulty in controlling the uniformity of the large-area microstructures and can only apply for small-area microstructures. Chou [12] proposed two types of roll-to-plate nanoimprinting methods, which can reduce the force between the mold and the polymer sheet, thereby improving the uniformity of the pressure distribution, and the accuracy of continuous replication of large-area microstructures. Guo [13] proposed the roll-to-roll nanoimprinting technology, resulting in continuous replication of the microstructure with small imprinting/demolding forces and uniform pressure. Lim [14] proposed a roll-to-roll direct nanoimprint lithography process, which can create nano/microstructures on a roll with a diameter of 250 mm and a width of 366 mm, to achieve the imprinting of nanostructured films.

Even though current imprinting methods for general optical sheet fabrication have achieved excellent results, there still exist difficulties in uniformity of microstructures and large depth-to-width ratio microstructures, especially for ultra-thin, wide-width, large-area optical sheets. Furthermore, existing imprinting molding technologies require polymer flat sheets as preform so that flat sheets need to be pre-created in advance and reheated before molding, which leads to a long production cycle and low production efficiency.

As a newly developed molding technology, injection-rolling can achieve high precision replication of large-area complex microstructures on the polymer surface by creatively combining injection molding and rolling [15,16]. To describe the temperature distribution of the injection-rolling zone more accurately, a novel temperature model was developed through a mathematical analysis method considering the influence of heat radiation, heat conduction and plastic deformation [17].

Different from conventional micro-imprinting methods, the polymer in the injection-rolling process changes from a viscous state to a rubbery state, and finally into a glass state, which means the finished product cannot be affected by the processing history of the raw materials, and the quality can be greatly improved. While, on the other hand, the state transition of the polymer in the roll gap will be more complicated, which means the effect of injection-rolling process parameters on the state transition of the polymer should be thoroughly investigated. Based on the finite element simulation and experimental methods, this paper studied the relationship between the state transition of the polymer and the injection-rolling process parameters, including injection temperature, roll temperature and roll rotational speed. Subsequently, the effect of the state transition interface positions on the forming mechanism of the microstructure was also investigated and discussed.

## 2. Experimental Method and Simulation

### 2.1. Setup and Process Parameters

**Figure 1:**
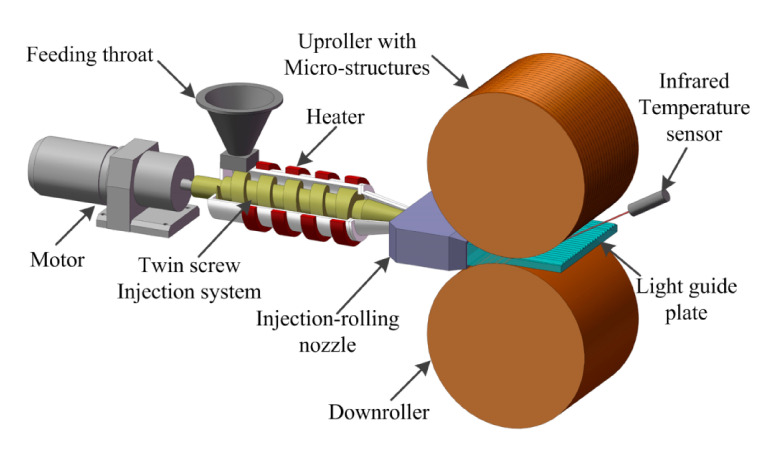
Schematic diagram of the injection-rolling molding process.

Figure 1 shows the schematic diagram of the injection-rolling process of the ultra-thin, large-area polymer optical sheets. The specific process is as follows: firstly, the polymer raw materials are dried, heated and melted into the viscous state, and then transported to the injection nozzle under the action of the twin-screw rotation and the melt pump. After being divided, compressed and insulated by the injection nozzle, the fluid polymer is then injected into the rolling mill at a uniform speed and temperature. Next, the microstructures on the roll surface will be filled by the fluid polymer and the fluid polymer continues to be transported forward under the rolling force of the two rolls. Due to the pressure of the rolls in the cooling stage, the fluid polymer gradually changes from the viscous state to the rubbery state, and finally to the glass state, resulting in forming a sheet with high-precision surface microstructures.

In the process of our basic experimental research, it was found that three process parameters, including the roll temperature, *T*_R_, the rolling speed, *V*_R_, and the injection temperature, *T*_0_, have a significant effect on controlling the injection-rolling process. Therefore, these three parameters are selected as variable parameters to characterize different injection-rolling process conditions in this paper. We found that all these parameters could affect product processing by affecting the interface position of the polymer state transition in the roll gap. When the interface is too far from the outlet, the sheet surface will be scratched, and even the polymer sheet may get stuck in the roll gap. Conversely, when the interface is too close to the outlet, the shape accuracy of the microstructure on the optical sheet will be reduced due to insufficient holding pressure or too-high outlet temperature. Both phenomena will degrade the performance and quality of the finished product, and even cause the interruption of continuous production. Therefore, interface position of the polymer state transition is the core to control the injection-rolling process, which affects the thermal equilibrium conditions in the polymer-forming process, the microstructure accuracy of the sheet and the stress and strain generated by the sheet deformation after complete solidification.

Based on previous experiments, the appropriate injection temperature range is ascertained as 200–240 °C and the rolling speed range of 5–25 mm/s was selected for the experiments according to the capacity of the injection-rolling equipment. In the simulation and experiments, the exit thickness of polymer sheet and the height of the injection nozzle were selected as 0.3 and 1 mm, respectively. The injection rolling machine was self-developed by Shenzhen University. For the extruder part, the power and the maximum extrusion pressure are 25 kW and 40 MPa, respectively. For the twin screw in the extruder, the diameter and the maximum speed are 16 mm and 500 r/min, respectively. The material used in the experiment is PMMA (polymethyl methacrylate) (CM205, CHIMEI, Tainan, Taiwan), which had been dried at 90 °C for four hours before the experiments.

### 2.2. Finite Element Modeling

**Figure 2:**
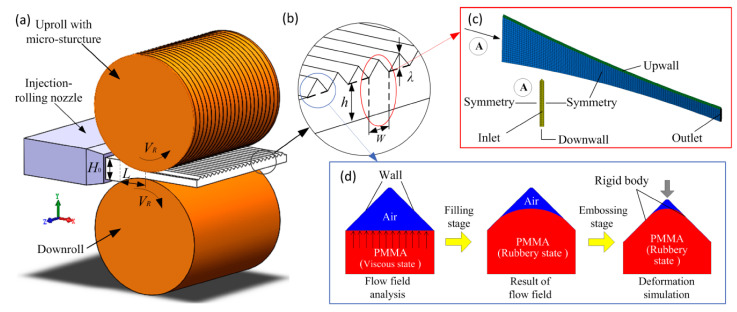
Geometric model and mesh model of the injection-rolling zone. (**a**,**b**) sketch map of injection-rolling zone, (**c**) finite element model of the injection-rolling zone, (**d**) finite element model of the microstructure-forming process.

The injection-rolling zone is the area that filled with polymer fluid from the injection nozzle to the roll gap between the upper roll and lower roll, as shown in Figure 2. It can be seen from the schematic diagram that the fabricated optical sheet has multiple repeating microstructures in the width direction. In order to facilitate the analysis, the temperature difference in the width direction of the sheet has been ignored, and the injection-rolling zone is simplified into multiple identical flow regions with the length of *L* and the width of *W*. Therefore, the three-dimensional model of the injection-rolling zone was established and divided into meshes using finite element software, as shown in Figure 2c.

During the injection-rolling process, as the PMMA fluid enters the microstructure and solidifies due to the heat exchange with the roll, the microstructure on the surface of the roll can be replicated to the optical sheet surface. To simulate this entire process, the process was divided into two stages, as shown in Figure 2d. The first stage is the filling of PMMA fluid into the microstructure. As the filling process goes on, the PMMA fluid gradually cools to the highly elastic state without any fluidity, where the filling process ends. The second stage is the imprinting of the highly elastic PMMA. The profile of PMMA material obtained in stage one is imported into the plastic deformation model for further analysis. In this stage, the roll gap gradually becomes smaller as the rolls rotate, which can be regarded as the microstructure mold pressing down and PMMA being further compressed, so the final shape can be obtained until all the PMMA transforms into glass state in the microstructure.

The temperature and flow fields of the polymer in the injection-rolling zone were numerically solved by ANSYS (ANSYS, Canonsburg, PA, USA) finite element analysis software. The rolls are rigid and cannot be deformed, and the rotation speeds of the upper and lower rolls are consistent. The heat and momentum transfer along the width of the roll are neglected, that is, the temperature distribution in the width direction is uniform. The injection-rolling was discretized using hexahedron elements, and there were 126,251 elements in the whole domain for solving the three-dimensional (3D) model in Figure 2c. The models in Figure 2d were simplified as plane models discretized with quadrilateral elements, and there were 10,945 and 9341 elements for solving flow field analysis and deformation simulation during the microstructure-forming process.

#### 2.2.1. Mathematical Models and Boundary Conditions

The continuous injection-rolling process combines the fluid dynamics, heat transfer and plastic deformation analysis. The governing equations for numerical simulation of polymer flow field in the injection-rolling zone are as follows:

(1) $$\rho \left(\frac{\partial H}{\partial t}+\nabla \cdot vH\right)=\nabla \cdot \left(k\nabla T\right)$$

(2) $$\frac{\partial \rho }{\partial t}+\nabla \cdot \left(\rho v\right)=0$$

(3) $$\frac{\partial }{\partial t}\left(\rho v\right)+\nabla \cdot \left(\rho vv\right)=-\nabla p+\nabla \cdot \left(\tau \right)+\rho g+F$$

where *ρ* is the density of polymer fluid, *H* is the enthalpy, *v* is the fluid velocity, *p* is the static pressure in the injection-rolling zone, *τ* is the stress tensor, *ρg* is the gravitational body force and *F* is the external body forces.

In the hot-pressing stage, the PMMA materials are set as thermo-compression elastoplastic deformation. The generalized Maxwell model is used to describe the viscoelastic behavior of the material, thereby characterizing the stress relaxation process and creep process of PMMA material [18,19], as shown in Equation (4):

(4) $$\sigma (t)=\varepsilon_{0}E_{e}+\sum_{i=1}^{n}E_{i}\exp(-\frac{t}{\tau_{i}})$$

where ε_0_ is the strain, *E*_e_ is the elastic modulus of the elastic element when *n* = 0, *E*_i_ is the *i*-th order elastic modulus and *τ* is the relaxation time.

The viscoelastic model in the finite element model is the general integral form of the Maxwell model, and its relaxation function is represented by the Prony series, as shown in Equation (5):

(5) $$E(t)=E_{0}+\sum_{i=1}^{n}E_{i}\exp(-\frac{t}{\tau_{i}})$$

In the flow field analysis of the injection rolling zone, the inlet can be formulated as,

*P*_x_ = *P*_in_, *P*_y_ = 0, *P*_z_ = 0, *T*_in_ = *T*_0_ (6)

where *P*_in_ is the component of inflow pressure in X direction and had a relationship with *V*_R_ in value that summarized by experiments as *P*_in_ = 1.72 *V*_R_ [15], *V*_R_ is linear velocity of roll and inflow temperature, *T*_in_, is equal to the initial temperature of polymer, *T*_0_.

Heat-transfer coefficient (HTC) is an important parameter to describe the thermal behavior of polymer during the rolling process. Contact heat transfer between the molten PMMA and the roll mold can be formulated as:

(7) $$q=h_{c}(T_{M}-T_{R})$$

where *q* is the heat flux, *h_c_* is the HTC, *T_M_* is the surface temperature of the polymer in the injection-rolling zone and *T_R_* is the surface temperature of the roll. So far, there are many theoretical and experimental studies that can obtain the value of HTC, but due to different material properties of the experimental instrument, processing parameters and surface roughness of the cavity wall, the value range of HTC is still large, which is in most cases between 1000 and 10,000 W/(m^2^ K) [20,21]. In this paper, according to the literature [22], the thermal conductivity between the roll and PMMA is set to 3000 W/(m^2^ K). The corresponding parameters of the simulations are shown in Table 1.

#### 2.2.2. Material Property

The material used in the model is the polymethyl methacrylate (PMMA) with a glass transition temperature of 105 °C and a viscous flow temperature of 160 °C (CM205, CHIMEI, Tainan, Taiwan). In the simulation model, the density, specific heat capacity and thermal conductivity of PMMA are 1180 kg·m^−3^, 1460 J·kg^−1^·K^−1^ and 0.19 W·m^−1^·K^−1^ [23,24]. The viscosities of PMMA fluid are 1102, 1045 and 855 Pa·s at 220, 230 and 240 °C respectively, measured by the polymer fluid rheology experiment [25].

## 3. Results and Discussion

### 3.1. Simulation Results and Experimental Verification

#### 3.1.1. Results and Verification of Injection-Rolling Zone Model

**Figure 3:**
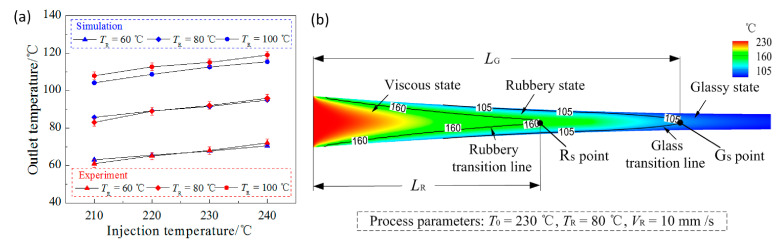
Simulation and experimental results of injection-rolling zone (**a**) verification of simulation model (**b**) temperature field result.

To evaluate the accuracy of the three-dimensional model established in this paper, the outlet temperature of the polymer in the injection-rolling process was calculated and then verified by the measured results. To measure the outlet temperature of the polymer, an infrared temperature sensor (T40-P7-30-SF0-1, Fluke Corporation, Everett, WA, USA) was used to detect the outlet temperature of the optical sheet surface, as clearly described in Figure 1. To obtain an accurate result, the experiment was performed five times and an average value was calculated as the final outlet temperature. The difference between the numerical calculation and experimental measurement of the outlet surface temperature is shown in Figure 3a, and the measurement error of the temperature sensor is below ±1 °C. Figure 3a illustrates the simulation and experiment comparison of the outlet temperatures under conditions of the rolling speed of 10 mm/s and injection temperatures of 210, 220, 230 and 240 °C. It can be seen from the figure that the outlet temperature of the PMMA sheet gradually increases with the initial temperature of the polymer and the roll temperature. The calculated steady state temperatures are both in good agreement with the measured values, and the simulation model is reliable and accurate.

Figure 3b shows the simulation result of the polymer temperature field and velocity field in the roll gap during the injection-rolling process. According to the state transition of the polymer, the injection-rolling zone can be divided into three regions in the horizontal direction, which are viscous state region (injection and filling), rubbery state region (condensation and heat transfer) and glass state region (pressure maintaining).

In the injection zone, the polymer surface contacts with the roll for rapid cooling, heat exchange and filling into the microstructure. As the rolls rotate, the fluid temperature continuously decreases and a rubbery state transition interface is formed, where the viscous flow polymer begins to gradually transform into rubbery state. When the injection-rolling process continues and the contact heat exchange with the roll, the sheet temperature gradually decreases, and the glass state transition interface is formed. It can be seen from Figure 3b that the profile of the polymer transition interface presents an approximate isosceles triangle shape, which makes it difficult to analyze the influence of the process parameters on the state transition mechanism of the polymer in the roll gap. For the convenience of analysis, we used the distance (*L*_R_ and *L*_G_) from the inlet of the injection-rolling zone to the end point of the polymer state transition interfaces (point *R*_s_ and point *G*_s_) to represent the interface position of the polymer state transition in the roll gap.

#### 3.1.2. Verification of Microstructure-Forming Model

**Figure 4:**
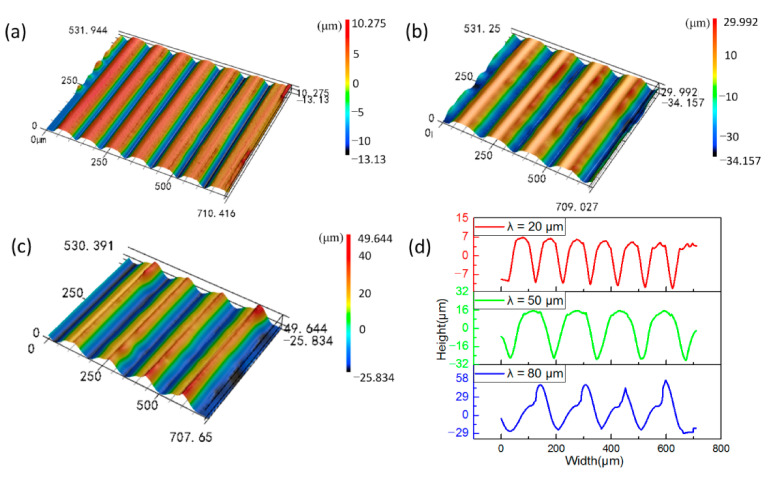
The profiles of microstructures with height of (**a**) λ = 20 μm, (**b**) λ = 50 μm, (**c**) λ = 80 μm, and (**d**) the measured data of microstructures.

The height and width of the microstructure of V-groove were measured with a Confocal laser scanning microscope (VK-250, KEYENCE, Osaka, Japan). The microstructure profile on a cross-section of the optical sheet is shown in Figure 4. The results indicate that, the average heights of the microstructures with λ = 20, λ = 50 and λ = 80 on the PMMA optical sheet are 16.73, 41.67 and 69.42 μm respectively, and the average widths of the microstructures of each size are 99.77, 151.67 and 163.41 μm, respectively. Interestingly, the microstructure width of each size has exceeded the design value, which might be explained by two reasons. On the one hand, the microstructure size of the roll is very small compared with the overall size of the roll, which increases the difficulty in roll machining. There may be a certain size error of roll in the microstructure at the place we measured. On the other hand, the excessive rolling pressure may be applied on the polymer in the injection-rolling process, causing an extra rolling reduction in the forming zone, thereby the size of the polymer microstructure may be larger than the size of the microstructure on the roll caused by springback when the rolling pressure no longer exerts on the sheet.

**Figure 5:**
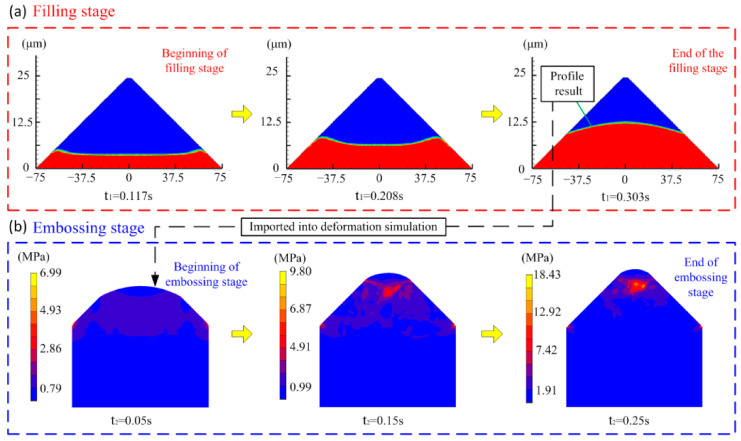
Simulation results of microstructure-forming process. (**a**) The results of flow analysis in microstructure filling stage, and (**b**) the results of deformation simulation in microstructure embossing stage.

According to the end point position of the polymer state transition interface obtained in Figure 3b, and parameters such as the roll speed and the length of the injection zone, the filling time, *t*_1_, and the imprinting time, *t*_2_, in the microstructure-forming process can be calculated. Figure 5a shows the calculation results of PMMA fluid filling in the microstructure at different *t*_1_. In the figure, the blue area is air, and the red area is PMMA fluid. As *t*_1_ increases, the filling height of the fluid in the microstructure gradually increases. When *t*_1_ = 0.533 s, PMMA has all been transformed into the highly elastic state, and the filling stage is over. Then, the final filling profile is imported into the imprinting model as the contour of the PMMA preform for further analysis. Figure 5b shows the deformation of the PMMA preform at different *t*_2_. When *t*_2_ = 0.25 s, the imprinting stage ends, and the final microstructure profile of PMMA can be obtained.

**Figure 6:**
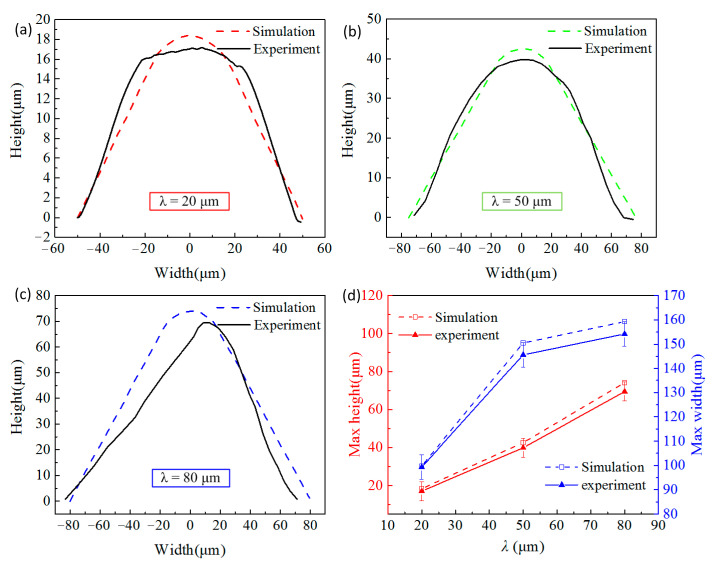
The microstructural profile comparison between experiment and simulation (**a**) λ = 20 μm, (**b**) λ = 50 μm, (**c**) λ = 80 μm, and (**d**) height and width comparison with different λ.

Figure 6 presents the comparison of the microstructure profile obtained by simulation and experiment under the same process conditions. It can be seen from the figure that the overall shape of the simulation result is very close to the experimental result when λ = 20 and 50 μm. While for λ = 80 μm, the simulated shape does not quite agree with the experimental data, even though the value of simulated height accords with the experimental height. Moreover, the height in the simulation result is larger than the experimental result and we speculate that the slight difference might result from PMMA material occurring springback after the injection-rolling process. Therefore, we can conclude that the simulation model used in this paper is feasible and accurate when microstructure height, λ, is in the range of 20–50 μm, while not applicable for accurately predicting microstructure height when λ is 80 μm, yet still provides instructions for microstructure forming at higher λ.

### 3.2. Effect of Microstructure Depth on Temperature Field in Injection-Rolling Zone

**Figure 7:**
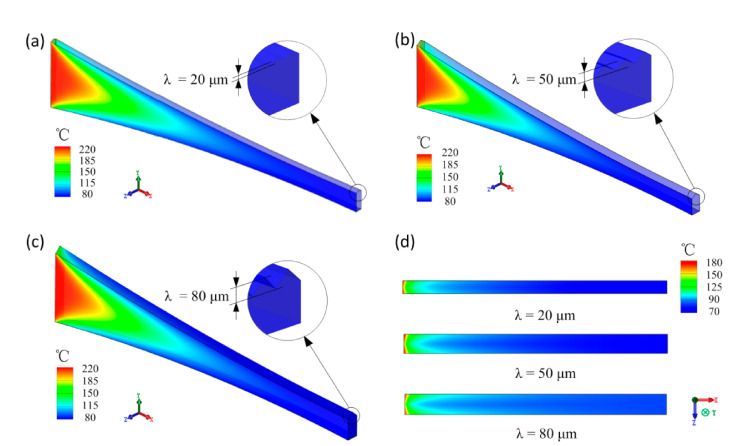
Temperature field of polymer in injection-rolling zone at different microstructure depths: (**a**) λ = 20 μm, (**b**) λ = 50 μm, (**c**) λ = 80 μm. (**d**) Temperature field of microstructure surface in injection-rolling zone.

The simulated horizontal position distribution and flow field of *R*_s_ and *G*_s_ in the injection-rolling zone are shown in Figure 4. The simulation setting is as follows: the injection temperature of polymer is 230 °C, the roll temperature is 80 °C and the rolling speed is 10 mm/s. Given that the aspect ratio of the injection-rolling zones is too tiny, the temperature contours in Figure 7a–c are enlarged by 2 times in the *y*-axis and *z*-axis directions, and in Figure 7d, the injection-rolling zone is enlarged by 5 times in the *z*-axis direction.

It can be seen from the simulation results that the presence of the microstructure has a certain effect on the temperature distribution of the polymer filled in the microstructure on the upper roll surface. When the injection-rolling process parameters are exactly the same, as the microstructure height of roll increases, the highest temperatures are 207.05, 206.6 and 207.18 °C, and the lowest temperatures are 80.38, 80.32 and 80.33 °C on the up-wall surface in Figure 7d. While, it can also be seen that the microstructure has a relatively small influence on the temperature distribution of the entire injection-rolling zone, and the interface positions of the state transition in the flow field with various microstructure depths are almost the same. Therefore, to simplify the following analysis, the influence of the microstructure on the temperature field distribution can be ignored, that is, the temperature field of the central cross-section of the model in the *z*-axis direction can be taken for analysis.

The effect of process parameters on the interface position of the state transition in the injection-rolling zone was investigated by analyzing the main process parameter variables (roll temperature, *T*_R_, injection temperature, *T*_0_, rolling speed, *V*_R_).

### 3.3. Effect of Parameters on Horizontal Positions of R_s_ and G_s_

**Figure 8:**
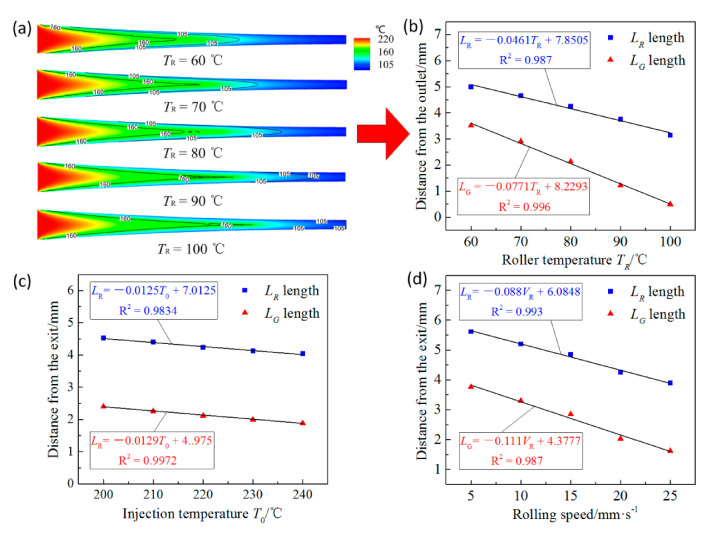
(**a**) Distributions of *R*_s_ and *G*_s_ in the injection-rolling zone at different roll temperatures, variation of *L*_R_ and *L*_G_ length with (**b**) roller temperature, (**c**) injection temperature, (**d**) rolling speed.

Under conditions that the injection temperature of the polymer and the rolling speed are set as 220 °C and 20 mm/s respectively, the horizontal positions of *R*_s_ and *G*_s_ in the injection-rolling zone at different roll temperatures are shown in Figure 8a. When other process parameters are constant, as the roll temperature increases, *L*_R_ and *L*_G_ gradually enlarge. This is because as the roll temperature increases, the temperature difference between the polymer and the roll surface decreases, and the heat transfer intensity between them gradually decreases, which slows down the cooling process of the polymer in the injection-rolling zone, making the positions of *R*_s_ and *G*_s_ move to the outlet as the roll temperature increases.

The lengths of *L*_R_ and *L*_G_ at different roll temperatures are shown in Figure 8b. It can be seen from the figure that as the roll temperature increases, *L*_R_ and *L*_G_ show the linear increase. Therefore, linear curves are fitted to obtain the relationship between *L*_R_ and *L*_G_ and roll temperature, which are presented in Figure 8b. The determination coefficients, *R*^2^, of fitted linear curves on *L*_R_ and *L*_G_ are 0.996 and 0.987, respectively.

Similarly, we can obtain the effects of *T*_0_ and *V*_R_ on *G*_s_ and *R*_s_ through simulation results. Under conditions that the roll temperature and the rolling speed are set as 80 °C and 20 mm/s respectively, the horizontal positions of *R*_s_ and *G*_s_ in the injection-rolling zone at different injection temperatures are shown in Figure 8c. When other process parameters are constant, as the injection temperature increases, the distances *L*_R_ and *L*_G_ gradually increase. The main reason is that as the injection temperature increases, more heat can be carried by the polymer, which leads to longer time for the polymer temperature to drop to the rubbery or glass state under the same roll temperature, thereby the state transition interface moves close to the outlet. Moreover, due to the large temperature difference and strong heat exchange between the roll and the initial polymer, the extra heat carried by the polymer will dissipate quickly. Therefore, linear curves are fitted to obtain the relationship between *L*_R_ and *L*_G_ and injection temperature, which are presented in Figure 8c. The determination coefficients, *R*^2^, of fitted linear curves on *L*_R_ and *L*_G_ are 0.997 and 0.983, respectively.

Under conditions that the injection temperature of the polymer and the roll temperature are set as 220 and 80 °C respectively, the horizontal positions of *R*_s_ and *G*_s_ in the injection-rolling zone at different rolling speeds are shown in Figure 8d. When other process parameters are constant, as the rolling speed increases, the distances *L*_R_ and *L*_G_ slightly increase. Therefore, linear curves are fitted to obtain the relationship between *L*_R_ and *L*_G_ and rolling speed, which are presented in Figure 8d. The determination coefficients, *R*^2^, of fitted linear curves on *L*_R_ and *L*_G_ are 0.987 and 0.993, respectively.

Based on the above analysis, it can be seen that in the injection-rolling process, the horizontal position of *R*_s_ and *G*_s_, on the interface from viscous flow state to rubbery state and on the interface from rubbery state to glass state, are approximately linearly affected by the injection temperature, the rolling speed and the roll temperature, respectively. According to Reference [26], the relationship between the process parameters and the position of the state transition points was obtained by fitting the simulation results, in order to predict the lengths of *L*_R_ and *L*_G_ under the changeful working conditions. The relationship between the lengths of *L*_R_ and *L*_G_ and the injection temperature, roll temperature and rolling speed can be deduced as Equations (8) and (9). The determined coefficients, *R*^2^, of Equations (8) and (9) are 0.977 and 0.984, respectively.

(8) $$L_{R}=0.028\times \left(0.0461T_{R}+2.3895\right)\times \left(0.0125T_{0}+3.2275\right)\times \left(0.088V_{R}+4.1552\right)$$

(9) $$L_{G}=0.015\times \left(0.0774T_{R}+1.9925\right)\times \left(0.0129T_{0}+5.265\right)\times \left(0.111V_{R}+5.8623\right)$$

### 3.4. Effect of State Transition Interface on Microstructure Filling Rate

As an important index to evaluate the quality of the microstructure on the optical sheet, the filling rate can be used to describe the replication accuracy of the injection-rolling process. The width and height of the microstructure were measured respectively, and the filling rate can be calculated according to Formula (10):

(10) $$\begin{array}{c} \eta_{\lambda }=\frac{\lambda^{\prime }}{\lambda }\\ \eta_{W}=\frac{W^{\prime }}{W} \end{array}$$

where *λ*′ and *W*′ are the measured height and width of microstructures, and *λ* and *W* are the height and width of microstructures on the roll surface.

To obtain the relationship between filling rate and position variation of state transition interface, the filling rates of the microstructures on the optical sheets under different processing parameters were measured. Five cross-sections are randomly selected to measure the depth of the microstructure, and the average value of the obtained data is used as an evaluation index.

**Figure 9:**
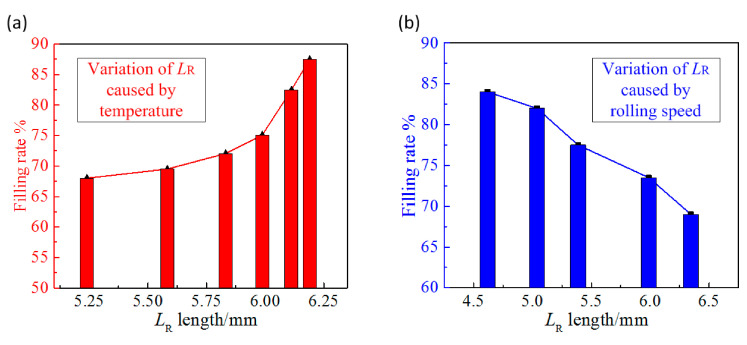
Variation of microstructure height with *L*_R_ caused by (**a**) temperature, (**b**) rolling speed.

Figure 9a shows the variation curve of microstructure depth with roll temperature or the injection temperature under conditions that the rolling speed is 20 mm/s. It can be seen from the figure that the microstructure filling rate increases with the increase of *L*_R_ length. The reason is that as the roll temperature or injection temperature increases, the position of the rubbery transition interface moves to the outlet, as shown in Figure 8b,c. Given that other injection-rolling parameters remain unchanged, when the length of viscous flow region becomes larger, the liquid polymer requires longer time to flow in the microstructure, which will lead to higher filling rate. Figure 9b shows the variation curve of filling rate with rolling speed under conditions that the roll temperature is 80 °C and the injection temperature is 230 °C. The filling rate microstructure depth decreases with the increase of the rolling speed. With the increase of the rolling speed, the flow speed of the polymer in the injection-rolling zone is accelerated and the filling time is shortened. Meanwhile, since the state transition interface moves to the outlet, the time for holding pressure on the polymer will be reduced after it is transformed into the rubbery state, resulting in a decrease in the filling depth of the microstructure.

We can conclude that the state transition interface moves to the outlet of the injection-rolling zone with the increase of the injection temperature and the roll temperature, and the filling rate of microstructure also increases. While, as the rolling speed increases, the state transition interface also moves toward the outlet, but the filling rate of the microstructure decreases accordingly. Therefore, the polymer in the injection-rolling process is both temperature-dependent and time-dependent, which means that the filling rate of the microstructure cannot be directly predicted by determining the position of the state transition interface without considering the rolling speed.

Within the range of process parameters, when the injection temperature and roll temperature are high, the polymer has better fluidity, which is conducive to the forming process. However, as the rolling speed increases, the high flow speed of the polymer leads to a reduction in the forming time, which is not conducive to the forming process. Therefore, the microstructure filling rate can only be predicted and adjusted by comprehensively considering the position of the transition interface and rolling speed.

### 3.5. Optimal Process Parameters for the Injection-Rolling Process

Theoretically, to achieve a high-replication process, the microstructures on the roll surface should be completely filled with the polymer fluid, which means the length of *L*_R_ should be sufficiently long for a complete filling. Also, the length of *L*_R_ should not be too long because sufficient time for hot embossing and pressure maintaining is needed for polymer to go through the following rubbery state. Therefore, we believe that point *R*_s_ located in the near center of the injection-rolling zone is appropriate. Meanwhile, *L*_G_ should be close to the outlet because the polymer will be in glass state at the outlet, which can not only improve the microstructure filling rate caused by demolding and elastic recovery, but also reduce the rolling pressure to ensure the accuracy of the shape and thickness difference.

**Figure 10:**
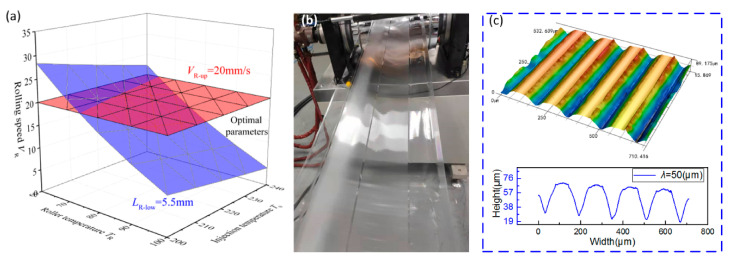
Optimal process parameters for the injection-rolling process: (**a**) optimal parameters, (**b**) pictures of injection-rolling process, with (**c**) microstructural measurement of the experiment.

It can be seen from Figure 9a that as *L*_R_ increases, the filling rate of microstructure gradually increases. Therefore, *L*_R_ and *L*_G_ should satisfy as *L*_R_ ≥ 0.55 *L* and *L*_G_ < *L,* where *L* is the length of the injection-rolling zone, as shown in Figure 2a, and *L* is 10.24 mm for the devices employed in this paper. So, the lower limit value of *L*_R_ (*L*_R-low_) and the upper limit value of *L*_G_ (*L*_G-up_) are 5.5 and 10.24 mm, respectively. In Figure 9b, as rolling speed increases, *L*_R_ increases correspondingly, leading to a drop in the filling rate of microstructure. Considering that forming time will also influence the process, the rolling speed should be selected at a lower value (*V*_R_ ≤ 20 mm/s) to improve the filling rate of the microstructure. In the range of *T*_R_ and *T*_0_ discussed in the paper, *L*_G_ < *L* can be satisfied when *V*_R_ ≤ 20 mm/s according to Equation (9). So, the range of process parameters is illustrated in Figure 10a as the space between red and blue areas. The optimal process parameters in Figure 10a can facilitate to achieve larger filling rate of microstructure rapidly in the experiments. In addition, filling rate of microstructure can be further improved by slightly adjusting process parameters based on the effect of parameters on the position of the state transition interface.

To further verify the optimized range of process parameters, a set of parameters (*T*_R_ = 90 °C, *T*_0_ = 210 °C, *V*_R_ = 15 mm/s) in the range was adopted to conduct the injection-rolling experiments and the achieved product is shown in Figure 10b. The surface morphology of the microstructures was measured, and it is illustrated in Figure 10c that the average height of microstructure with λ = 50 μm is 41.25 μm, leading to a high filling rate.

## 4. Conclusions

(1) This paper established a thermal-flow coupling model for the injection-rolling zone. The effect of various process parameters on the high elastic transition point, *R*_s_, glass transition point, *G*_s_, and flow field of the injection-rolling zone were analyzed in detail. The polymer state transition interface presents isosceles triangle distribution in the injection-rolling zone.

(2) The analysis for the effect of parameters on the transition state of the polymer in the roll gap indicates that point *R*_s_ and point *G*_s_ move toward the outlet of the injection rolling zone with the increase of injection temperature, roll temperature and rolling speed. For the injection-rolling equipment in this paper, the position of the state transition interface in the injection-rolling zone can be predicted from the process parameters according to Equations (8) and (9), which provides theoretical guidance for subsequent injection-rolling experiments of optical sheets.

(3) The effect of the state transition interface position on the filling rate of the microstructure and the reason for the effect were revealed by analyzing the microstructure size of the optical sheet under different injection-rolling conditions. The position of the rubbery state transition interface greatly influences the flow time of the polymer in the filling stage, thereby affecting the filling rate of microstructures. Therefore, in future research, the quality of the optical sheet can be further improved by adjusting the position of the polymer state transition interface in the injection-rolling zone.

## Figures and Tables

**Table 1:** Simulation parameters.

Parameters	Unit	Value
Roll diameter, *R*	mm	300
Initial thickness *H*_0_/Outlet thickness, *h*	mm	1/0.3
Microstructure height, λ/width, *W*	μm	20/100, 50/150, 80/160
Initial temperature, *T*_0_	°C	200, 210, 220, 230, 240
Roll temperature, *T*_R_	°C	60, 70, 80, 90, 100
Rolling speed, *V*_R_	mm/s	10, 15, 20, 25

## Data Availability

Data available in a publicly accessible repository.
